# Portal Vein Thrombosis in Cirrhotic Candidates for Liver Transplantation and Its Impact on the Transplant Accessibility

**DOI:** 10.3390/jcm15093358

**Published:** 2026-04-28

**Authors:** Simona Parisse, Flaminia Ferri, Adriano De Santis, Fabio Melandro, Mario Corona, Quirino Lai, Pierleone Lucatelli, Gianluca Mennini, Massimo Rossi, Stefano Ginanni Corradini

**Affiliations:** 1 Wellbeing, Health and Environmental Sustainability, Sapienza University of Rome, Via della Fontanelle, 02100 Rieti, Italy; simona.parisse@uniroma1.it (S.P.); adriano.desantis@uniroma1.it (A.D.S.); 2 AOU Policlinico Umberto I, Viale del Policlinico 155, 00161 Rome, Italy; 3 Department of Translational and Precision Medicine, Sapienza University of Rome, AOU Policlinico Umberto I, 00161 Rome, Italy; flaminia.ferri@uniroma1.it; 4 General Surgery and Organ Transplantation Unit, Department of General and Specialty Surgery, Sapienza University of Rome, AOU Policlinico Umberto I, 00161 Rome, Italy; fabio.melandro@uniroma1.it (F.M.); quirino.lai@uniroma1.it (Q.L.); gianluca.mennini@uniroma1.it (G.M.); massimo.rossi@uniroma1.it (M.R.); 5 Vascular and Interventional Radiology Unit, Department of Radiological Oncological and Anatomo-Pathological Sciences, AOU Policlinico Umberto I, 00161 Rome, Italy; mario.corona@uniroma1.it (M.C.); pierleone.lucatelli@gmail.com (P.L.)

**Keywords:** liver transplant, portal vein thrombosis, cirrhosis, obesity, MASLD

## Abstract

**Background/Objectives**: Portal vein thrombosis (PVT) is prevalent among candidates for liver transplantation (LT) and may serve as a contraindication to transplantation when extensive. Given the rising prevalence of metabolic syndrome, this study aimed to identify clinical factors associated with PVT and evaluate its impact on access to the LT waiting list and the likelihood of undergoing transplantation. **Methods**: A retrospective cohort of 711 consecutive patients assessed for LT between 2008 and 2020 was included. Data on PVT and various clinical variables were collected, including reasons for exclusion from the waiting list and dropout rates. Multivariable logistic regression models with forward selection and bootstrap were constructed to assess factors associated with PVT, access to the waiting list, and LT. **Results**: PVT was identified in 11.6% of patients (*n* = 83), with advanced thrombosis observed in 21% of cases. Obesity emerged as the only independent factor significantly associated with the presence of PVT (*p* = 0.001, OR 2.619, 95% CI 1.577–4.352). Patients with PVT were more frequently excluded from the waiting list due to clinical contraindications compared to those without PVT (26% vs. 14%, *p* = 0.04). However, multivariable analysis did not demonstrate an independent association between PVT and access to the waiting list or LT. No significant differences were observed in the reasons for dropout from the waiting list between patients with and without PVT. **Conclusions**: PVT appears to be associated with the metabolic profile of LT candidates, particularly obesity; however, it does not significantly limit access to LT.

## 1. Introduction

Portal vein thrombosis (PVT) is a complication that may occur in patients with liver cirrhosis as a consequence of portal hypertension. It is more frequently observed in individuals with advanced liver disease, characterized by high MELD or Child–Pugh scores, acute decompensation, clinically significant portal hypertension, as well as in patients with hepatocellular carcinoma (HCC) [1,2,3,4]. Both conditions represent well-established indications for liver transplantation (LT) [5]. Accordingly, PVT is commonly encountered among LT candidates, although its reported incidence varies widely, ranging from approximately 5% to 26% with a reported pooled prevalence in liver cirrhosis of pooled prevalence of 13.9% [4,6,7].

It is well established that, when present, PVT has a significant impact on outcomes among candidates for LT. In particular, PVT increases the technical complexity of the transplant procedure, and it remains debated whether extensive thrombosis (e.g., Yerdel grade 3–4) may even be considered a relative contraindication to transplantation [7,8,9,10,11]. Moreover, extensive PVT has been associated with reduced post-transplant survival of both the recipient and the graft [10,11,12]. Nevertheless, the currently available evidence suggests that LT in patients with PVT still provides a survival benefit and should not be regarded as futile [9,13,14]. Therefore, its presence has substantial clinical relevance in potential LT candidates, as it may affect both access to transplantation and post-transplant success.

Among the clinical risk factors associated with PVT, male sex, cirrhosis due to metabolic dysfunction-associated steatotic liver disease (MASLD), and selected features of the metabolic syndrome have been reported as significant [15,16,17,18]. In particular, several studies have described significant associations between PVT and a high body mass index (BMI), the latter assessed both as a continuous variable and by categorizing patients as obese or non-obese [18,19,20].

However, although much of the available evidence suggests a robust association between PVT and MASLD-related cirrhosis, the relationship between PVT and individual components of the metabolic syndrome, especially obesity, remains controversial and has not been consistently confirmed or explored across studies [21,22,23]. This uncertainty is further highlighted by the findings of a relatively recent meta-analysis [24]. Nevertheless, precise identification of the major risk factors for PVT is of paramount importance, particularly given the increasing prevalence of metabolic syndrome and MASLD among LT candidates, to enable the implementation of targeted clinical strategies [25,26].

Although several studies have examined the impact of PVT on post-transplant morbidity and mortality and have explored predictors of post-transplant outcomes, the effect of PVT across the entire LT candidate pathway, from waitlist access to transplantation, has not yet been comprehensively investigated.

For this reason, we conducted a single-center study to investigate the prevalence of PVT among LT candidates across the entire transplant evaluation pathway, from the initiation of transplant assessment to transplantation. Specifically, we aimed to: (1) identify clinical factors associated with the presence of PVT in LT candidates; (2) assess whether and how PVT influences access to the LT waiting list; and (3) evaluate the impact of PVT on the likelihood of undergoing LT among listed patients.

## 2. Materials and Methods

### 2.1. Study Design

This was a retrospective monocenter observational study investigating data of cirrhotic patients referred to LT according to the presence or absence of PVT. The present study was conducted in accordance with the Declaration of Helsinki and was approved by the Local Ethics Board of Sapienza University of Rome (Ref. N. 233 October 2002 and Ref N.420. 27 November 2014). The Strengthening the Reporting of Observational Studies in Epidemiology (STROBE) guidelines were followed to create this study.

### 2.2. Study Population

Consecutive adult cirrhotic patients (age > 18) with indication to LT between 2008 and 2020 were considered eligible for inclusion in the study. The indication for LT was determined based on the current rules of the LT Italian program, based on current national and international recommendations [5,27].

Exclusion criteria included: pediatric age, age > 70 years, the presence of tumor-related PVT at baseline or during follow-up, fulminant hepatitis, acute on chronic liver failure (ACLF), and absence of an indication for LT.

### 2.3. Portal Vein Thrombosis Assessment

All patients included in the study were managed according to the local protocol for screening of PVT. This protocol includes a baseline contrast-enhanced abdominal computed tomography (CT) scan at the initiation of the transplant evaluation process, followed by Doppler ultrasound surveillance every 6 months prior to placement on the waiting list.

After listing for LT, patients underwent abdominal CT annually and Doppler ultrasound every 3 months. In patients with confirmed or suspected HCC, radiological follow-up with CT or magnetic resonance imaging (MRI) was performed according to clinical indication.

When a Doppler ultrasound detected PVT or raised suspicion of thrombosis, based on a significant reduction in portal flow velocity and/or flow inversion in the portal vein, a contrast-enhanced abdominal CT scan was performed to confirm the diagnosis and to assess the stage and extent of thrombosis. PVT was diagnosed based on the presence of intraluminal material within the portal vein consistent with non-tumoral thrombosis on contrast-enhanced CT.

When PVT was diagnosed, staging was assessed at baseline and classified according to the Yerdel classification system in Multidisciplinary Team Discussion [8] Four PVT grades were therefore identified: Grade 1 (G1): partial PVT with <50% luminal occlusion; Grade 2 (G2): >50% luminal occlusion, including complete occlusion of the portal vein, with or without minimal extension into the superior mesenteric vein (SMV); Grade 3 (G3): complete thrombosis of the portal vein and the proximal SMV; Grade 4 (G4): complete thrombosis of the portal vein and both proximal and distal segments of the SMV. Cases of intrahepatic non-tumoral PVT (classified as G0) were also considered, given their potential risk of extension during follow-up and the need for specific therapeutic evaluations.

When PVT was identified, cases were discussed in a multidisciplinary team meeting to determine the most appropriate therapeutic strategy, taking into account both PVT characteristics and the patient’s clinical profile. Management options included anticoagulation alone, anticoagulation combined with transjugular intrahepatic portosystemic shunt (TIPS), TIPS alone in patients with contraindications to anticoagulation, or conservative management in patients with unfavorable clinical conditions.

### 2.4. Data Collection

The clinical and demographic characteristics of each patient were retrieved from outpatient and hospitalization records. Particular attention was given to the presence of comorbidities associated with metabolic syndrome, particularly obesity, which was defined as a body mass index (BMI) ≥ 30 kg/m, and the etiology of liver cirrhosis, with a specific focus on the presence of metabolic dysfunction-associated steatotic liver disease (MASLD). MASLD was diagnosed based on the presence of at least two of the following criteria: (1) BMI > 25, (2) diabetes, (3) dyslipidemia, or (4) arterial hypertension [28,29].

Patients were followed throughout the entire LT evaluation process. For each patient, data were collected regarding potential inclusion on the LT waiting list or, alternatively, the reasons for non-listing. Among patients who were wait-listed, additional information was collected on access to LT or the reasons for removal from the waiting list.

All clinical outcomes occurring during both the LT evaluation phase and the waiting-list period were systematically recorded and classified into the following categories: (a) death; (b) clinical contraindications; (c) HCC-related contraindications; (d) loss to follow-up; and (e) clinical improvement. Clinical contraindications were assessed during the transplant evaluation process and, as recommended, included the presence of significant comorbidities (such as metabolic, cardiovascular, pulmonary, neurological, or psychiatric conditions) with a predicted 5-year mortality >50%, impaired performance status, inadequate social support, and active or previous extrahepatic malignancies not meeting transplant criteria [5,30,31].

### 2.5. Statistical Analysis

The Kolmogorov–Smirnov test was applied to assess the distribution of variables, revealing that all continuous variables included in the study did not have a normal distribution. Therefore, continuous variables were reported as medians with interquartile ranges (IQR), while categorical variables were presented as counts and percentages. There were no missing data, so imputation methods were not required. Group differences were evaluated using the Mann–Whitney U test for continuous variables and the chi-square (χ^2^) or Fisher’s exact test for categorical variables.

Several binary logistic regression models were constructed, with clinically relevant variables selected as covariates for each model. In detail, the binary logistic regression analysis for factors associated with the presence of PVT was constructed using two separate models to avoid collinearity among the variables. The first model included variables related to metabolic syndrome, while the second incorporated clinical and demographic variables, including MASLD. In subsequent binary logistic regression models, access to the waiting list for LT and access to LT were considered as dependent variables. Clinico-demographic variables deemed clinically relevant were included in the models, with PVT entered as a covariate. Covariates were selected to balance analytical completeness with minimizing type I error. Forward stepwise elimination was used to refine the models, ensuring only significant predictors were retained. Bootstrapping techniques were employed to enhance robustness and predictive accuracy.

Collinearity testing confirmed no significant collinearity among covariates. All collinearity tolerance coefficients exceeded the threshold of 0.1, indicating no significant multicollinearity. Linearity between continuous independent variables and their log odds was verified using the Box-Tidwell transformation test. Extreme outliers were assessed using Cook’s distance, and logistic regression results showed no substantial changes when analyses were repeated after excluding the few identified outliers.

All statistical tests were two-tailed, with a significance level set at *p* < 0.05. Analyses were conducted using SPSS version 25.0 (SPSS Inc., Chicago, IL, USA), ensuring rigorous and accurate statistical interpretation.

## 3. Results

**Figure 1:**
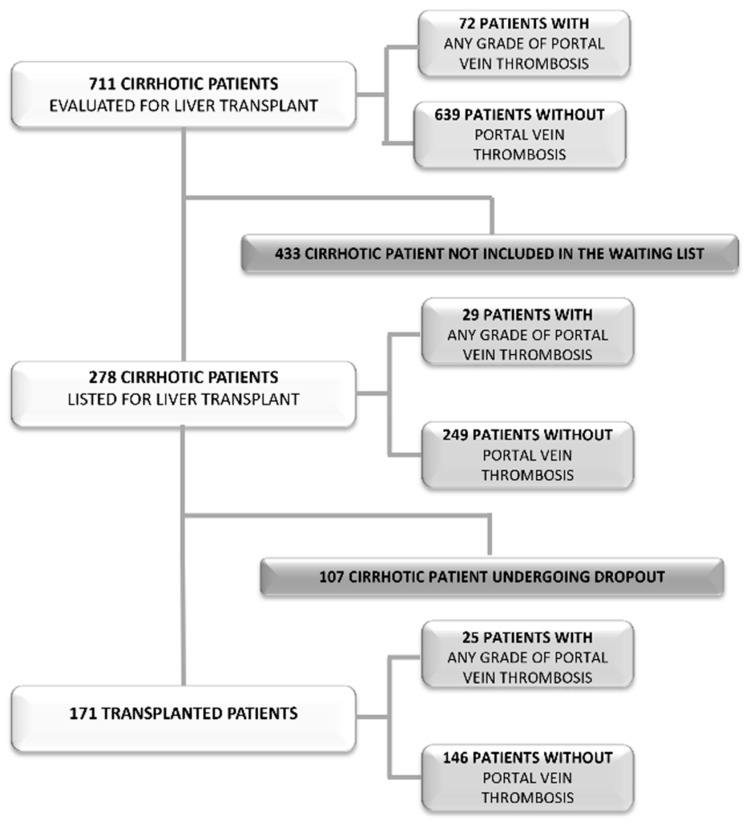
This figure schematically illustrates the study population at the different stages of the liver transplant evaluation process and the corresponding distribution of PVT. The number of patients with PVT represents the number of patients diagnosed with PVT of any grade at the respective time points of observation.

Between 2008 and 2020, a total of 711 patients with cirrhosis were evaluated for LT at the Transplant Center of the Policlinico Umberto I, Sapienza University of Rome. Of these, 83 patients (11.6%) were diagnosed with PVT of any grade during the LT evaluation process. Specifically, 72 cases of PVT were identified before inclusion on the LT waiting list, whereas 11 cases were diagnosed after listing. The following analyses compare patients with and without PVT at different time points along the transplant evaluation pathway. Accordingly, the PVT groups include patients diagnosed with thrombosis during the pre-listing evaluation, at the time of waitlist inclusion, or during the waiting-list period up to transplantation. Figure 1 shows the study population at the different stages of the LT evaluation process and the corresponding distribution of PVT.

A detailed evaluation of the extent of PVT revealed exclusively intrahepatic involvement in 18% of cases (*n* = 15) and Yerdel grades 1, 2, 3 and 4 in 48% (*n* = 40), 13% (*n* = 11), 16% (*n* = 13) and 5% (*n* = 4) of cases, respectively.

Regarding management strategies, 59% of patients with PVT (*n* = 49) received at least one therapeutic intervention. Among these, 71.4% (*n* = 35) were treated with anticoagulation alone, 12.2% (*n* = 6) underwent TIPS placement, and 16.3% (*n* = 8) received a combination of TIPS and anticoagulant therapy. Among the 34 patients who did not receive any treatment, approximately one third (*n* = 10) had exclusively intrahepatic PVT, while the remainder (*n* = 24) were ineligible for treatment due to clinical contraindications, high bleeding risk, or advanced-stage PVT.

**Figure 2:**
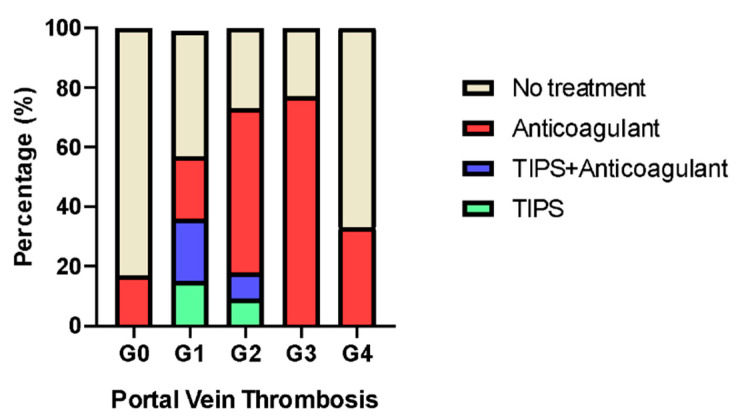
Distribution of treatment strategy according to portal vein thrombosis stage among potential liver transplant candidates.

Figure 2 displays the distribution of treatment strategies according to the stage of PVT.

### 3.1. Factors Associated with Portal Vein Thrombosis Among Liver Transplant Candidates

Comparison of clinical and demographic characteristics between patients with and without PVT revealed that the only statistically significant differences were observed in body mass index (BMI) and the prevalence of obesity (Table 1). Notably, obesity was more than twice as prevalent among patients who developed PVT at any stage of the LT pathway compared with those who did not develop PVT. Normal-weight patients accounted for 43% of patients with PVT and 52% of those without PVT. Regarding other components of metabolic syndrome, such as dyslipidemia, arterial hypertension, and diabetes, these conditions were more frequently observed in the PVT group; however, the differences did not reach statistical significance. No significant differences were identified between the two groups with respect to liver disease severity, underlying etiology, or the presence of HCC.

We subsequently investigated whether any independent associations existed between clinical variables and the presence of PVT within our cohort. As shown in Table 2, we constructed multivariable binary logistic regression models. The first model included only demographic and metabolic variables. The principal finding was that obesity was independently and significantly associated with the presence of PVT, increasing its likelihood by more than 2.5-fold. To further explore the association with obesity, we repeated the multivariable analysis using BMI as a continuous exposure variable instead of the dichotomous obesity variable. In this model, higher BMI remained significantly associated with PVT diagnosed at any stage of the LT pathway (OR 1.066, 95% CI 1.012–1.124; *p* = 0.031). Similarly, when BMI was categorized into three groups (normal weight, overweight, and obesity), the association remained statistically significant (OR 1.527, 95% CI 1.178–2.098; *p* = 0.002).

In the second model, we evaluated variables primarily related to liver disease. Interestingly, MASLD did not exhibit a significant association with PVT. Conversely, alcohol-related liver disease showed a significant inverse association with the occurrence of PVT (Model 2, Table 2).

### 3.2. Factors Associated with Inclusion in the Waiting List for Liver Transplantation and Reasons for Being Excluded from Listing in the Entire Study Population and According to the Presence of Portal Vein Thrombosis

A total of 278 patients were included on the LT waiting list; among these, 29 (10%) had a diagnosis of PVT at the time of listing. Of the 711 patients evaluated for LT, 433 (60%) were not listed. As detailed in Table 3, statistically significant differences emerged between listed and non-listed patients. In particular, MASLD was more frequently identified as the underlying etiology among listed patients, which was paralleled by a higher prevalence of diabetes and dyslipidemia. In contrast, alcohol-related liver disease was more common among patients who were not listed. Notably, HCC was associated with a higher likelihood of listing, representing 50% of all patients placed on the waiting list. No significant differences were observed between the two groups in terms of obesity or the presence of PVT. Within the groups of patients with and without PVT at the time of listing, the differences between listed and non-listed patients remained largely consistent with those reported in Table 3, particularly among patients without PVT. In patients with PVT, however, the only statistically significant differences were observed for obesity (48% in listed vs. 23% in non-listed patients, *p* = 0.04) and alcohol-related etiology (20% in listed vs. 46% in non-listed patients, *p* = 0.03) (Supplementary Table S1).

To further explore predictors of access to the LT waiting list, we developed a multivariable binary logistic regression model, with inclusion on the LT waiting list as the dependent variable. As reported in Table 4, PVT at the time of listing did not retain statistical significance in relation to listing eligibility and was removed during the forward selection process. In contrast, HCC, MELDNa score, and MASLD emerged as independent predictors significantly increasing the likelihood of waitlist access. Conversely, alcohol-related liver disease was significantly associated with reduced odds of listing.

**Figure 3:**
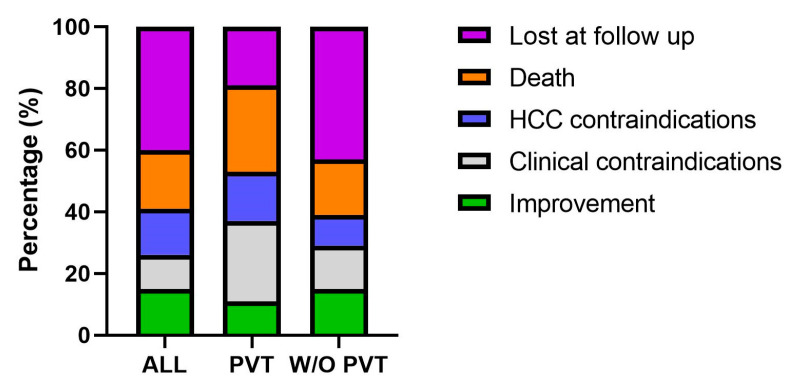
Reasons for not reaching the liver transplant waiting list in the entire population, in patients with PVT and in patients without PVT.

Lastly, when examining the reasons for failure to access the LT waiting list in patients with and without PVT, we found that individuals with PVT diagnosed during the pre-listing evaluation for LT were more frequently excluded due to the presence of clinical contraindications (*p* = 0.04) and were less likely to be lost to follow-up compared to those without PVT (*p* = 0.003). No significant differences were observed between the two groups in terms of clinical improvement, mortality, or contraindications related to HCC (Figure 3).

### 3.3. Factors Associated with Access to Liver Transplantation and Reasons of Dropout from the Waiting List in Listed Patients and According to the Presence of Portal Vein Thrombosis

In the final part of our study, we evaluated access to LT among patients who were placed on the waiting list. Among the 278 listed patients, 61% (*n* = 171) underwent LT.

When comparing transplanted and non-transplanted patients, it is notable that male sex and the presence of HCC were significantly more frequent in the transplanted group (Table 5). No statistically significant differences were observed between the two groups in terms of liver disease etiology, comorbidities, or the presence of PVT, either at the time of listing or diagnosed during the waiting-list period.

As in previous sections, we sought to explore whether independent associations existed between clinical variables and the likelihood of undergoing transplantation. When constructing the binary logistic regression model with LT as the dependent outcome, using the same covariates included in the previous model (see Table 4), PVT again did not demonstrate a significant association with LT eligibility. In this model, HCC was the only variable that remained statistically significant (*p* = 0.015; OR 1.917, 95% CI 1.183–3.106).

Lastly, no meaningful differences were observed between transplanted and non-transplanted patients according to PVT status during the waiting-list period (Supplementary Table S2). Similarly, when examining the reasons for failure to undergo LT, no statistically significant differences emerged between patients with and without PVT in terms of waiting list dropout (Supplementary Figure S1).

## 4. Discussion

In the present study, we investigated the clinical determinants of PVT and evaluated its impact across the entire LT candidate pathway, from transplant evaluation through the waiting-list period to transplantation. To our knowledge, few studies have assessed PVT across the entire LT trajectory.

In our cohort, PVT was observed in 11.6% of patients, a prevalence consistent with previously published studies in the transplant and non-transplant setting [4,6,7]. Although all patients evaluated for LT were included, those with advanced PVT (Yerdel grade 3–4) were poorly represented, accounting for only approximately one fifth of patients with PVT. This underrepresentation is likely attributable to pre-enrollment referral bias, as only a limited number of patients with advanced PVT may have been considered eligible for LT and consequently referred to our transplant center for evaluation. Regarding clinical management, approximately two-thirds of patients with PVT referred to our center, regardless of whether they were ultimately placed on the LT waiting list, received specific treatment aimed at limiting thrombus progression and facilitating access to transplantation.

The main finding of our study is that obesity emerged as the primary clinical determinant independently associated with PVT. This result is particularly relevant and consistent with previous studies reporting a similar association [18,19,20].

Obesity is widely recognized as a major and growing public health concern, with an increasing prevalence in the general population and, consequently, among patients with cirrhosis evaluated for liver transplantation, raising significant clinical and ethical issues [32,33,34,35]. Data from United States registries indicate that in 2023, approximately 40% of patients undergoing LT were obese. Furthermore, given the dynamic nature of body weight and obesity over time, recent evidence has shown that longitudinal increases in body weight significantly influence the progression of MASLD and the risk of liver-related events [36]. Therefore, when evaluating patients with chronic liver disease, it may be important to consider not only the impact of obesity itself on clinical outcomes, but also the potential effects of weight changes over time, including both weight gain and weight loss. In this context, it can be hypothesized that increases in body weight over time may be associated with a higher risk of developing PVT. All these findings highlight the growing burden of obesity-related complications in the hepatology field and consequently in the transplant setting [37].

In our study, approximately one fifth of obese candidates for LT in our cohort were affected by PVT. This observation is plausible, as increasing evidence suggests that obesity is associated with a pro-inflammatory and prothrombotic state. Obese individuals exhibit increased levels of prothrombotic factors, including D-dimer, fibrinogen, factor VIII, and factor IX. In addition, a well-documented state of chronic low-grade systemic inflammation, characterized by elevated levels of C-reactive protein (CRP), may further promote a hypercoagulable condition [38,39,40].

In the setting of cirrhosis and LT candidates, most available studies have reported differences in BMI or obesity status between patients with and without PVT. In particular, consistent with our findings, Ayala et al. identified obesity as a risk factor for PVT in multivariate analysis in a cohort of patients undergoing LT [19]. Similarly, another study conducted in a large cohort of LT recipients showed, in univariate analysis, that patients with PVT had a higher BMI and a greater prevalence of obesity [20]. Partially overlapping findings were reported by Molinari et al., who observed an independent association between BMI and PVT; however, obesity itself was not included in the multivariate model. In their univariate analysis, the association between obesity and PVT appeared variable: only class I obesity was more frequent among patients with PVT, whereas class II obesity was paradoxically more prevalent among patients without PVT, and no differences were observed in individuals with class III obesity [18]. Conversely, other studies failed to demonstrate statistically significant differences in BMI and/or obesity prevalence between patients with and without PVT, making this association controversial [22,23]. These discrepancies may partly explain why a significant association has not been consistently demonstrated in meta-analyses [24].

An additional confounding factor is the technical difficulty of ultrasonographic assessment. Although ultrasound is generally considered accurate for the diagnosis of PVT, it should be noted that this technique may have limitations in obese patients. This may lead to underdiagnosis of PVT, particularly when thrombosis is limited in extent [41,42,43]. In this regard, the diagnostic protocol adopted at our center likely minimizes this bias, allowing a more accurate estimation of PVT prevalence in obese individuals. Indeed, all LT candidates undergo careful ultrasonographic monitoring performed by experienced sonographers within a dedicated liver imaging service, in addition to periodic abdominal computed tomography scans.

Contrary to previously reported data, MASLD-related cirrhosis was not significantly associated with PVT in our study [15,16,17,18,20]. A possible explanation for this finding may lie in the heterogeneity of the MASLD population, which includes patients with different combinations of metabolic syndrome components that may differentially influence the prothrombotic state.

In contrast, alcohol-related cirrhosis was found to be protective against PVT, in line with prior findings reported by Fan et al. [44]. Possible explanations include both the clinical improvement and lower risk of hepatic decompensation observed in LT candidates with alcoholic cirrhosis, who are required to maintain abstinence in order to access transplantation, as well as the hypocoagulable profile described in this patient population [44,45].

An additional relevant finding of our study concerns the impact of PVT along the LT pathway in cirrhotic patients. In multivariable analyses, PVT was not significantly associated with key steps of the transplant process. Instead, the factors independently associated with inclusion on the waiting list and with subsequent transplantation were major demographic variables, age for waitlist inclusion and sex for access to transplantation, and clinical characteristics, including MASLD etiology, alcohol-related liver disease, HCC, and MELD-Na score for waitlist inclusion, and HCC for access to LT.

These findings support the notion that, although PVT represents a clinically relevant condition that may influence therapeutic decision-making and requires careful multidisciplinary management, its presence does not appear to directly impair access to LT. It is plausible that close clinical and imaging surveillance, together with appropriate therapeutic management in transplant candidates with PVT, allows continuation of the transplant pathway without a statistically significant reduction in access to LT. Moreover, advances in surgical techniques have likely contributed to overcoming what was previously considered a major technical barrier to transplantation.

However, when analyzing the reasons for non-inclusion on the waiting list, patients with PVT were more frequently excluded due to clinical contraindications compared with those without PVT, even though PVT is well known to be associated with acute decompensation, higher MELD scores, and HCC [1,2,3]. This finding supports the concept already reported in the literature that PVT is not an absolute contraindication to LT but may influence the transplant eligibility assessment and contribute to the decision-making process regarding candidate suitability. In addition, several factors commonly considered when evaluating transplant eligibility include cardiometabolic characteristics, comorbidities, and other extrahepatic conditions, which are more frequently observed in obese patients [46]. Obesity itself, although not regarded as an absolute contraindication to LT, is nonetheless taken into account during the transplant evaluation process [30]. Overall, it is therefore plausible that patients with PVT in our cohort had a higher burden of comorbidities and were consequently more often excluded from the waiting list. Interestingly, patients with PVT were less frequently lost to follow-up. This may reflect greater disease awareness and closer clinical monitoring, likely related to the need for more frequent imaging assessments and specialized care in this patient population.

Several limitations should be acknowledged. This was a retrospective, single-center study conducted on a relatively limited sample size, and the results may have been influenced by selection bias, particularly due to the relatively low representation of patients with advanced PVT. An additional limitation of the present study is the inability to evaluate the impact of spontaneous or treatment-related temporal changes in the severity of PVT throughout the LT evaluation pathway. In particular, it was not possible to analyze in detail the effect of different PVT treatment strategies on the extent of thrombosis over time. This limitation is mainly attributable to the relatively small number of patients with PVT, the heterogeneity of the treatments administered, and differences in patients’ clinical conditions. Furthermore, graft and post-transplant patient survival could not be analyzed; therefore, we were unable to investigate whether and how PVT influenced post-transplant outcomes or whether this could also be related to the clinical characteristics of patients with PVT, who in our cohort appeared to have a higher prevalence of obesity. Moreover, we were unable to investigate individual thrombophilic predisposition or to explore potential associations between the severity of portal hypertension and the presence of PVT. Larger prospective studies are therefore warranted to confirm and expand these findings, which currently support the notion that, although PVT appears to be associated with the metabolic profile of LT candidates, it does not significantly limit access to LT.

## 5. Conclusions

In conclusion, PVT is a relatively common condition among patients with cirrhosis who are candidates for LT and is significantly associated with a more complex metabolic profile, characterized by obesity and elevated BMI. Although PVT was not significantly associated with the likelihood of being placed on the transplant waiting list or undergoing transplantation, our results indicate that patients with PVT are more frequently excluded from the waiting list due to clinical contraindications. This finding may be explained by the fact that, in our cohort, PVT was associated with obesity, and these patients more often presented with multiple comorbidities that, collectively, may influence the assessment of eligibility for liver transplantation.

Our findings are consistent with previous literature suggesting an association between obesity and PVT and further highlight the increasing complexity of LT candidates in the context of the rising prevalence of metabolic syndrome and obesity in this population. However, these results should be confirmed in larger cohorts and prospective studies to better investigate the risk factors associated with the development of PVT and to evaluate how changes in these factors over time may influence both the occurrence of PVT and patient prognosis, from a precision medicine and prevention perspective.

## Figures and Tables

**Table 1:** Clinical and demographic characteristics of the entire population divided according to the presence or absence of portal vein thrombosis.

Variable	Patients W/O PVT *n* = 628	Patients with PVT *n* = 83	*p* Value
Median (IQR) or *n* (%)			
Age	55.9 (49.4–61.2)	57 (50.3–61.8)	0.53
Male sex	496 (79)	65 (78)	0.89
MELD	14 (11–17)	15 (12–17)	0.51
MELDNa	15 (12–19)	16 (13–19)	0.40
BMI	25 (23–28.4)	27.2 (23.1–30.8)	0.03
Aetiology of liver disease:			
MASLD	184 (29)	29 (35)	0.31
Alcohol	301 (48)	31 (37)	0.08
HCV	233 (37)	24 (29)	0.15
HBV	79 (13)	12 (14)	0.60
CHO	39 (6)	4 (5)	0.80
HCC	240 (38)	39 (47)	0.15
Obesity	94 (15)	27 (32)	<0.0001
Overweight	200 (32)	20 (25)	
Normal weight	334 (53)	36 (43)	
Dyslipidemia	40 (6)	10 (12)	0.07
Arterial Hypertension	140 (22)	20 (24)	0.80
Diabetes	161 (26)	29 (35)	0.09

Abbreviations: BMI, body mass index; CHO, Cholestatic and/or Autoimmune cirrhosis; IQR, interquartile range; HCC, hepatocellular carcinoma; MASLD, Metabolic Dysfunction-Associated Steatotic Liver Disease; MELD, Model of End-Stage Liver Disease; MELDNa, Model of End-Stage Liver Disease Sodium; PVT, portal vein thrombosis; W/O, without. The number of patients with PVT represents the number of patients diagnosed with PVT of any grade during the entire period of observation. Obesity was defined as a body mass index (BMI) > 30 kg/m^2^, overweight as a BMI 25.0–29.9 kg/m^2^, and normal weight as a BMI 18.5–24.9 kg/m^2^.

**Table 2:** Multivariate binary logistic regression for presence of portal vein thrombosis.

		O.R.	95% CI		*p*-Value
			Lower	Upper	
Model 1	Obesity	2.619	1.577	4.352	0.001
Model 2	Age	1.000	0.975	1.025	0.971
	MELDNa	1.027	0.982	1.074	0.205
	HCC	1.656	0.972	2.824	0.069
	MASLD	1.067	0.638	1.784	0.779
	Alcohol	0.601	0.371	0.974	0.037
	HCV	0.623	0.369	1.052	0.083

Model 1 Age, Sex, Dyslipidemia, Arterial Hypertension, Type 2 Diabetes eliminated by the forward method. Abbreviations: CI, confidence interval; HCC, hepatocellular carcinoma; HCV, hepatitis C Virus; MASLD, Metabolic Dysfunction-Associated Steatotic Liver Disease; MELDNa, Model for End-stage Liver Disease Sodium; O.R., odds ratio.

**Table 3:** Clinical and demographic characteristics of the study cohort divided into listed and not listed patients.

Variable	Not Listed Patients *n* = 433	Listed Patients *n* = 278	*p*-Value
Median (IQR) or *n* (%)			
Age	56.8 (50.4–62.5)	55.8 (49.7–60.5)	0.20
Male sex	340 (78)	221 (79)	0.78
MELD	14 (11–17)	15 (11.3–18)	0.07
MELDNa	16 (12–19)	16 (12–20)	0.52
BMI	25 (22.9–28.7)	25.6 (23.1–28.7)	0.38
HCC	139 (32)	140 (50)	<0.001
Aetiology of liver disease:			
MASLD	104 (24)	109 (39)	<0.001
Alcohol	225 (52)	107 (38)	0.001
HCV	160 (37)	97 (35)	0.63
HBV	51 (12)	40 (14)	0.30
CHO	28 (6)	15 (5)	0.63
Obesity	67 (15)	57 (20)	0.105
Dyslipidemia	19 (4)	31 (11)	0.001
Arterial Hypertension	87 (20)	73 (26)	0.05
Diabetes	93 (21)	97 (35)	<0.001
Portal vein thrombosis	43 (10)	29 (10)	0.90

Abbreviations: CHO, Cholestatic and/or Autoimmune cirrhosis; HCC, hepatocellular carcinoma; MASLD, Metabolic Dysfunction-Associated Steatotic Liver Disease; MELD, Model of End-Stage Liver Disease; MELDNa, Model of End-Stage Liver Disease Sodium. The number of patients with PVT represents the number of patients diagnosed with PVT at listing.

**Table 4:** Multivariate binary logistic regression for access to liver transplant waiting list.

	O.R.	95% CI		*p*-Value
		Lower	Upper	
Age	0.972	0.955	0.989	0.002
MELDNa	1.043	1.010	1.077	0.017
HCC	2.781	1.922	4.024	0.001
MASLD	2.015	1.419	2.862	0.001
Alcohol	0.577	0.417	0.798	0.001

Sex and Portal Vein Thrombosis (PVT) eliminated by the forward method. Abbreviations: CI, confidence interval; HCC, hepatocellular carcinoma; MASLD, Metabolic Dysfunction-Associated Steatotic Liver Disease; MELDNa, Model for End-stage Liver Disease Sodium; O.R, odds ratio.

**Table 5:** Clinical and demographic characteristics of patients listed for liver transplant divided into transplanted and not transplanted.

Variable	Not Transplanted *n* = 117	Transplanted *n* = 171	*p* Value
Median (IQR) or *n* (%)			
Age	54.9 (49.7–59.9)	56.4 (49.5–60.9)	0.27
Male sex	85 (73)	136 (80)	0.02
MELD	15 (12–19)	14 (11–18)	0.34
MELDNa	16 (12–20)	15 (12–19.8)	0.39
BMI	25.3 (23.5–28.4)	25.6 (22.7–29.49)	0.82
HCC	48 (41)	92 (54)	0.01
Aetiology of liver disease:			
MASLD	44 (38)	65 (38)	0.70
Alcohol	52 (44)	55 (32)	0.08
HCV	42 (36)	55 (32)	0.79
HBV	13 (11)	27 (16)	0.23
CHO	10 (9)	5 (3)	0.06
Obesity	18 (15)	39 (23)	0.09
Dyslipidemia	12 (10)	19 (11)	0.70
Arterial Hypertension	29 (25)	44 (26)	0.68
Diabetes	36 (30)	61 (36)	0.25
Portal vein thrombosis	15 (13)	25 (15)	0.60

Abbreviations: CHO, Cholestatic and/or Autoimmune cirrhosis; IQR, interquartile range; HCC, hepatocellular carcinoma; MASLD, Metabolic Dysfunction-Associated Steatotic Liver Disease; MELD, Model of End-Stage Liver Disease; MELDNa, Model of End-Stage Liver Disease Sodium; The number of patients with PVT represents the number of patients diagnosed with PVT of any grade liver transplant.

## Data Availability

Data are available upon request to the corresponding author.

## Supplementary Materials

The following supporting information can be downloaded at https://www.mdpi.com/article/10.3390/jcm15093358/s1. Figure S1: Reasons for not reaching liver transplantation among all listed patients, among listed patients with PVT, and among listed patients without PVT; Table S1: Clinical and demographic characteristics of patients with and without PVT divided in listed and not listed patients; Table S2: Clinical and demographic characteristics of patients with and without PVT divided in transplanted and not transplanted.

## Abbreviations

The following abbreviations are used in this manuscript

BMI	Body mass index
CI	Confidence interval
CT	Computer tomography
HCC	Hepatocellular carcinoma
HCV	Hepatits C Virus
LT	Liver transplantation
MASLD	Metabolic Dysfunction-Associated Steatotic Liver Disease
OR	Odds ratio
PVT	Portal vein thrombosis
SMV	Superior mesenteric vein
TIPS	Tranjugular intrahepatic portosystemic shunt
US	Ultrasound
