# Supplementary material for: Portal Vein Thrombosis in Cirrhotic Candidates for Liver Transplantation and Its Impact on the Transplant Accessibility
Source: J Clin Med. 2026 Apr 28;15(9):3358. doi: 10.3390/jcm15093358 (PMC13163374; doi:10.3390/jcm15093358)
Supplement: Supplementary file 1 [file jcm-15-03358-s001.zip › Supplementary tables pvt 15-03-2026.pdf]

Supplementary Table S1. Clinical and demographic characteristics of patients with and without PVT divided in listed and not listed patients

**Variable**	**Patients W/O PVT N=639**			**Patients with PVT N=72**		
	**Not Listed N=390**	**Listed N=243**	**P Value**	**Not Listed N=43**	**Listed N=29**	**P value**
Age	55.6(50-62.4)	55.7(49.4-60.3)	0.26	58.3(52.8-63.6)	55.8(50.2-61.6)	0.58
Male sex	307(79)	197(81)	0.92	33(76)	24(83)	0.77
MELD	14(11-17)	15(11-19)	0.11	13(10.4-17)	15(13-17)	0.31
MELDNa	16(12-19)	16(12-20)	0.61	15(13-18.6)	16(13.5-20)	0.60
BMI	25(22.9-28.4)	25.1(23-28.4)	0.60	26(23-30.5)	29.4(24.9-31)	0.30
Aetiology of liver disease:						
MASLD	93(24)	95(39)	**0.001**	11(26)	14(48)	0.08
Alcohol	205(52)	101(42)	**0.004**	20(46)	6(20)	**0.03**
HCV	148(38)	90(37)	0.74	12(28)	7(24)	0.72
HBV	46(12)	34(14)	0.54	5(12)	6(20)	0.30
CHO	25(6)	15(6)	0.86	3(7)	0(0)	0.15
Obesity	57(15)	43(18)	0.43	10(23)	14(48)	**0.04**
Dyslipidemia	16(4)	25(10)	**0.004**	3(7)	6(20)	0.08
Arterial Hypertension	76(19)	66(27)	**0.032**	11(26)	7(24)	0.89
Diabetes	81(20)	84(34)	**<0.001**	12(28)	13(45)	0.20
HCC	124(32)	124(51)	**<0.001**	15(35)	16(55)	0.09
Yerdel						0.39
≤ 2	-	-	N/A	35(81)	21(72)	
3-4	-	-		8(19)	8(28)	

Abbreviations: CHO, Cholestatic and/or Autoimmune cirrhosis; HCC, hepatocellular carcinoma; LT, Liver Transplant; MASLD, Metabolic Associated Steatotic Liver Disease; MELD, Model of End Stage Liver Disease; MELDNa, Model of End Stage Liver Disease Sodium; LT, Liver transplant, TIPS, Transjugular Intrahepatic portosystemic shunt.

Supplementary Table S2. Clinical and demographic characteristics of patients with and without PVT divided in transplanted and not transplanted

**Variable**	**Patients W/O PVT N=238**			**Patients with PVT N=40**		
	**Not transplanted N=102**	**Transplanted N=136**	**P Value**	**Not transplanted N=15**	**Transplanted N=25**	**P value**
Age	54.4(49.5-60)	56.4(49.8-60.8)	0.44	55.5(49.7-58.4)	58.3(48-62.9)	0.29
Male sex	74(73)	115	0.04	11(73)	21(84)	0.44
MELD	14.5(11.5-19)	14(11-18)	0.49	16(15-18)	14(13-17)	0.21
MELDNa	16(12-20)	15(11.4-19)	0.45	17(15-19)	16(13.5-21)	0.58
BMI	25(23.5-28.1)	25.2(22.7-28.4)	0.83	25.7(24.1-30)	29.7(21.4-32)	0.32
Aetiology of liver						
disease:	38(37)	53(39)	0.89	6(40)	12(48)	0.75
MASLD	47(46)	49(36)	0.14	5(33)	6(24)	0.72
Alcohol	39(38)	46(34)	0.49	3(20)	9(36)	0.29
HCV	10(10)	23(17)	0.13	3(20)	4(16)	0.75
HBV	10(10)	4(3)	**0.02**	0(0)	1(4)	0.43
CHO						
Obesity	14(14)	26(19)	0.29	4(27)	13(52)	0.12
Dyslipidemia	9(9)	15(11)	0.67	3(20)	4(16)	0.75
Arterial Hypertension	26(25)	38(28)	0.77	3(20)	6(24)	0.77
Diabetes	30(29)	50(37)	0.27	6(40)	11(44)	0.80
HCC	41(40)	75(55)	0.03	7(47)	17(68)	0.20

Abbreviations: CHO, Cholestatic and/or Autoimmune cirrhosis; HCC, hepatocellular carcinoma; LT, Liver Transplant; MASLD, Metabolic Associated Steatotic Liver Disease; MELD, Model of End Stage Liver Disease; MELDNa, Model of End Stage Liver Disease Sodium; LT, Liver transplant, TIPS, Transjugular Intrahepatic portosystemic shunt.
