# Supplementary figures and images for: Portal Vein Thrombosis in Cirrhotic Candidates for Liver Transplantation and Its Impact on the Transplant Accessibility
Source: J Clin Med. 2026 Apr 28;15(9):3358. doi: 10.3390/jcm15093358 (PMC13163374; doi:10.3390/jcm15093358)

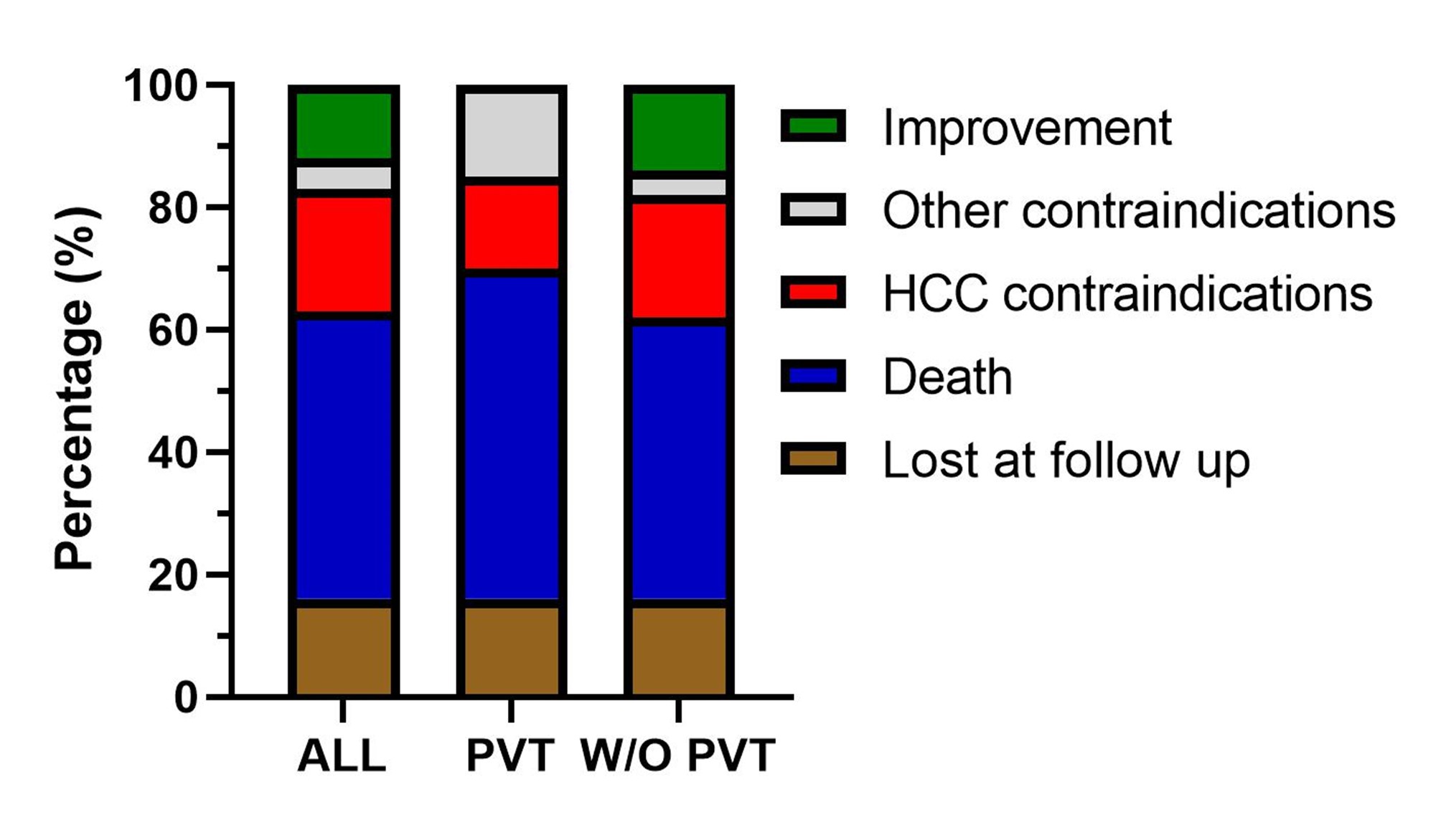

Supplement: Supplementary file 1 [file jcm-15-03358-s001.zip › Supplementary Figure S1.jpg]
